# Room-Temperature Self-Healing Polyurethanes Containing Halloysite Clay with Enhanced Mechanical Properties

**DOI:** 10.3390/polym17202807

**Published:** 2025-10-21

**Authors:** Eva Dauder-Bosch, José Miguel Martín-Martínez

**Affiliations:** 1 Noxun Adhesives, Scientific Park of Alicante, 03005 Alicante, Spain; 2 Adhesion and Adhesives Laboratory, University of Alicante, 03080 Alicante, Spain

**Keywords:** polyurethane, intrinsic room-temperature self-healing, halloysite, polycarbonate diol polyol, soft segments, mechanical reinforcement, filler–polymer interactions

## Abstract

Room-temperature self-healing polyurethanes (PUs) generally show limited mechanical properties. In order to improve the mechanical properties of PUs without sacrificing their self-healing ability, in this study, different amounts of halloysite clay filler were added. Thus, intrinsically self-healing PUs were synthesized using polycarbonate diol polyol, aliphatic diisocyanate, 1,4-butanediol, and different amounts (0.5–10 wt.%) of thermally treated halloysite. During synthesis, the halloysite clay was added to the polyol. The structural, thermal, viscoelastic, and mechanical properties of the resulting halloysite-filled PUs were evaluated. All halloysite-filled PUs retained their room-temperature self-healing capability while exhibiting improved mechanical strength. The PU with 0.5 wt.% halloysite (E0.5) showed the most balanced performance, with well-dispersed halloysite nanotubes intercalated within the soft segments, enhancing chain mobility and soft segment ordering. Higher halloysite loadings (1–3 wt.%) led to increased mechanical properties but also some round clay particle agglomeration and surface migration, leading to limited halloysite–polyurethane interactions. The addition of more than 3 wt.% halloysite did not result in further improvements in mechanical properties. The findings of this study provide new insight into the filler–polymer interaction mechanism and establish a foundation for the design of multifunctional PUs with both autonomous self-repair and enhanced mechanical performance.

## 1. Introduction

There is a growing interest in the development of self-healing polymeric materials for innovative applications in biomedical devices, coatings, structural adhesives, and flexible electronics, applications in which long-term durability and damage tolerance are critical. Self-healing polyurethanes (PUs) are very versatile materials due to their adaptable chemical architecture and thermal stability [1,2].

**Figure 1:**
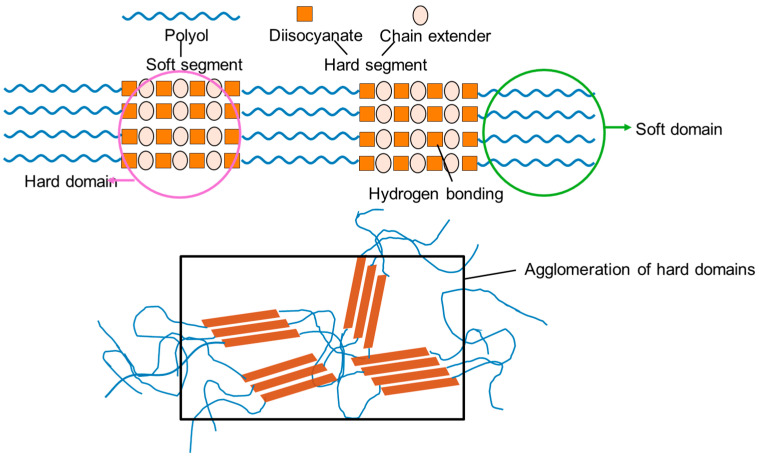
Phase-separated morphology in segmented polyurethanes (PUs), highlighting the organization of the soft and hard segments/domains.

PUs are typically synthesized via polyaddition reactions between polyols and diisocyanates, sometimes incorporating chain extenders. Their structure consists of alternating soft and hard segments. The soft segments are formed by high-molecular-weight polyol chains, which impart elasticity and chain mobility; the hard segments result from the reaction of isocyanates with low-molecular-weight diols or diamines and provide mechanical strength [3,4,5,6]. Due to the inherent polarity contrast between these segments, micro-phase separation occurs during PU synthesis. This phase-segregated morphology leads to amorphous or semi-crystalline hard domains dispersed in a soft, flexible matrix (Figure 1). The resulting architecture offers a unique combination of flexibility and structural integrity, with the hard domains acting as reversible physical crosslinks [5,6,7,8].

The micro-phase separation in PUs is further promoted by the formation of reversible hydrogen bonds between urethane and urea groups in the polymer network [8]. These reversible interactions not only contribute to the mechanical reinforcement of the polymer but also allow for a thermally and dynamically reconfigurable structure, which is essential for self-healing [9].

**Figure 2:**
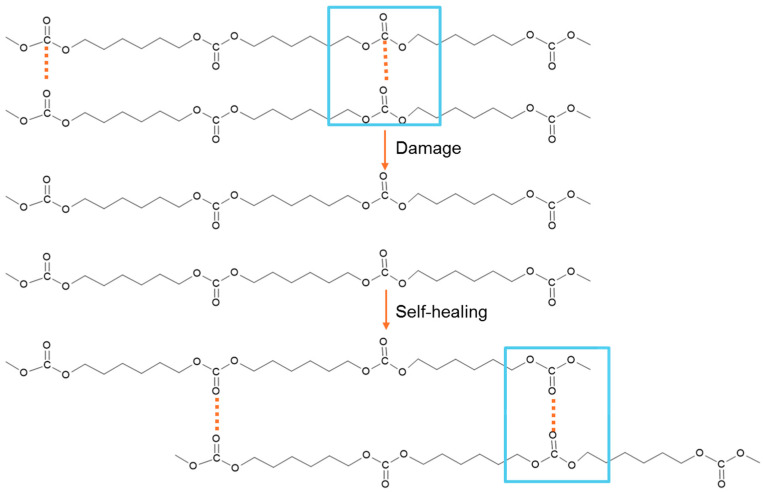
Non-covalent dynamic exchange room-temperature self-healing mechanism between polycarbonate soft segments in polyurethanes. The broken and re-formed interactions between carbonate groups in the soft segments are marked in blue boxes.

Among the wide variety of polyols, polycarbonate diol polyols (PCDs) have attracted attention as reactants in PU synthesis due to their superior hydrolytic and oxidative stability as well as their sustainability, since they can be derived from CO_2_ [8,10]. PUs synthesized with PCDs demonstrate an intrinsic room-temperature self-healing capability [1,11], which has been attributed to the dynamic non-covalent exchange interactions between carbonate groups in the soft segments (Figure 2) [1,11,12]. These interactions hold the supramolecular networks together, enabling reversible rearrangements after mechanical damage [1,13].

**Figure 3:**
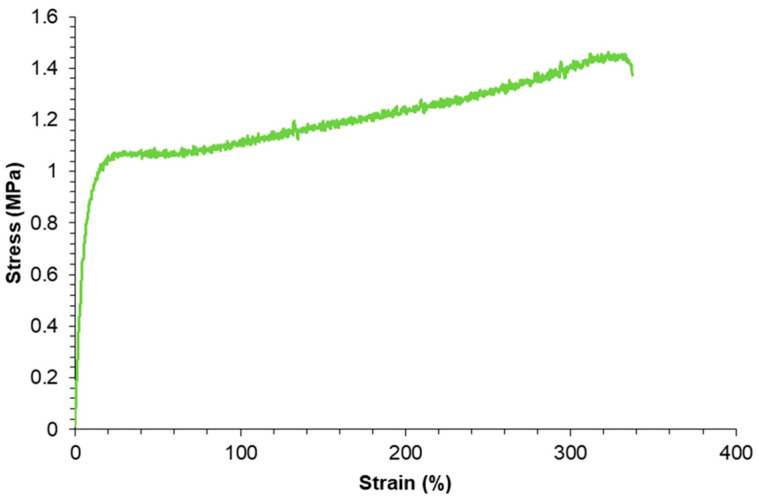
Stress–strain curve of room-temperature self-healing polyurethane made with polycarbonate diol polyol.

However, the intrinsic room-temperature self-healing capability of PUs synthesized with PCDs comes at the expense of reduced mechanical performance (Figure 3), including low tensile strength, a low yield point, and a low Young’s modulus, due to the mobility of the soft segments [11,12,13,14,15,16]. This results in a trade-off between self-healing efficiency and mechanical robustness, which currently limits the implementation of room-temperature self-healing PUs synthesized with PCDs in high-performance applications.

To overcome the limited mechanical performance of room-temperature self-healing PUs, PUs made with PCDs with a molecular weight of 500, 1000, and 2000 Da were synthesized [11]. Although the increase in the PCD molecular weight increased the mechanical properties of the PUs, a complete loss of the room-temperature self-healing ability was produced. On the other hand, PUs have been made with different blends of PCDs with molecular weights of 1000 and 2000 Da [17]. The increase in the PCD with a molecular weight of 2000 Da in the blend slowed the kinetics and increased the self-healing times of the PUs, but somewhat high tensile strength values (2–3 MPa) and moderate elongation-at-break values (150–200%) were obtained. Even though an increase in the mechanical properties was obtained, it was still insufficient for some demanding applications. Therefore, in this study, a different strategy consisting in the addition of fillers during the synthesis of self-healing PUs made with PCDs was considered.

The incorporation of fillers such as silicas and silicates, carbonates, carbon materials (carbon black, graphene, graphite), and clays has been commonly used to improve the mechanical properties, thermal stability, and wear resistance of PUs [18,19,20,21,22,23,24,25,26,27,28,29]. Silica fillers enhanced the viscosity, viscoelastic properties, and mechanical properties of PUs [20]. On the other hand, adding just 5 wt.% precipitated CaCO_3_ to PUs significantly increased the Shore A hardness and thermal degradation temperature [24]. Furthermore, silicate-based fillers, such as silica aerogels, and layered clays have been widely studied for reinforcing polyurethane matrices, i.e., significantly improving both the mechanical strength and thermal insulation. Their efficacy stems from the interaction with the polymer matrix via intercalation or exfoliation, particularly in nanoclays [21,26]. For instance, Lee et al. [22] demonstrated that adding 1–5 wt.% silane-modified silica aerogels to a rigid polyurethane foam improved the compressive strength and reduced the thermal conductivity. In a recent study, Babar et al. [28] reported that incorporating a combination of silica and nanoclay into thermoplastic polyurethane acted synergistically by leading to a thermal conductivity reduction of up to 40%, alongside notable improvements in the elastic modulus and impact resistance. On the other hand, the addition of carbon materials improved the mechanical and conductivity properties of PUs [17] and adhesion [19]. One recent study [29] reported that the self-healing properties of PUs were enhanced by multiple hydrogen bonds from ureido-pyrimidinone containing 1–3 wt.% graphene with improved mechanical properties. Self-healing was produced by heating at 80 °C for 1 h and reached 88–91% efficiency.

**Figure 4:**
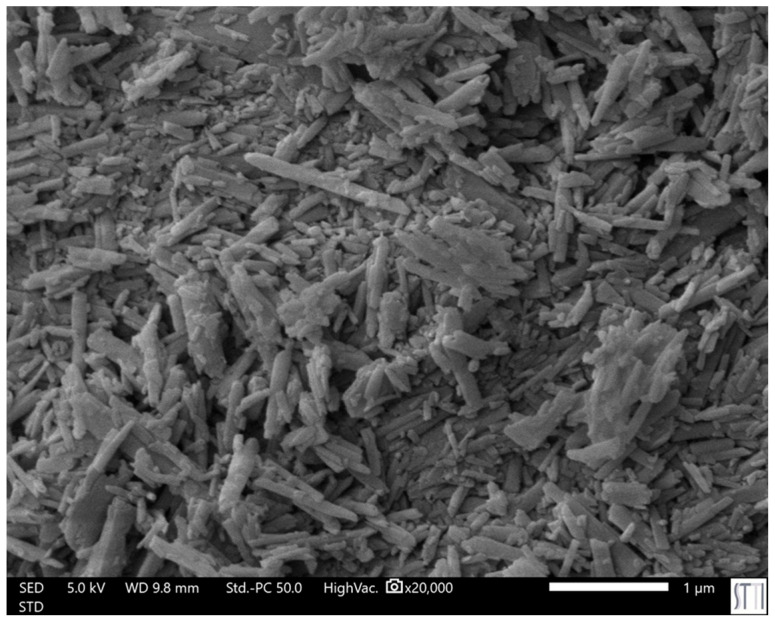
SEM micrograph of the thermally treated halloysite used in this study.

Naturally occurring halloysite clay has emerged as a promising candidate for increasing the mechanical properties of self-healing PUs. Halloysite is a biocompatible bilaminar aluminosilicate of the kaolinite group with the empirical formula Al_2_Si_2_O_5_(OH)_4_·nH_2_O. Halloysite exhibits a unique tubular morphology with a high aspect ratio (Figure 4), a large specific surface area, and excellent thermal and mechanical stability [30,31,32]. From a structural standpoint, the outer surface of halloysite nanotubes is composed mainly of siloxane (Si–O–Si) groups, while the inner lumen is rich in aluminol (Al–OH) groups; thus, its reactivity is due to Al–OH and Si–OH groups in defects on the surface and at the outer edges. The abundant hydroxyl groups on the halloysite surface favor the dispersion into the polymer matrix in comparison with other nanofillers [33], particularly in segmented PUs containing polycarbonate or polyether-rich soft segments [28,34]. Furthermore, the potential for nanodomain dispersion of halloysite contributes not only to mechanical reinforcement but also to modulation of phase separation, preserving the dynamic architecture essential for self-healing [35,36].

Previous studies have shown that the addition of up to 2 wt.% halloysite causes a significant improvement in the mechanical and thermal properties of PUs [37,38], which was ascribed to a reduction in their spherulite sizes [39]. The modification of halloysite with acids favored the interfacial compatibility between the halloysite and the PU, leading to better dispersion and enhanced mechanical properties [38,40]. More recently, the impact of adding 2–10 wt.% halloysite on the mechanical and shape memory properties of additively manufactured PUs was addressed [41]. The optimal mechanical properties were obtained in PU containing 8 wt.% halloysite, but the increased amount of halloysite inhibited the shape memory property.

To the best of our knowledge, there are very few studies on thermally self-healing PUs incorporating halloysite via in situ polymerization and reversible Diels–Alder intermolecular interactions [40,42,43,44,45]. These studies used ultrasonically treated halloysite in an organic solvent that was reacted with isocyanate. Afterwards, the isocyanate-grafted halloysite was reacted with different chemicals (furfuryl alcohol, polytetrahydrofuran polyol, etc.) to obtain a prepolymer that was crosslinked with different chain extenders (hexamethylene diisocyanate trimer, N-hydroxyethyl maleimide, etc.). All PUs containing halloysite showed good mechanical properties, but the self-healing of the halloysite-filled PUs was completed at 120 °C for 10 min and at 65 °C for 24 h.

Considering the above findings, there is a need to develop room-temperature self-healing PUs with improved mechanical properties. The addition of halloysite has been shown to be efficient at increasing the mechanical properties of PUs, and the previous studies on halloysite-filled self-healing PUs show a complex synthesis procedure and that self-healing is reached only by heating at 65–120 °C [40,42,43,44,45]. Considering that the PUs synthesized with PCDs exhibited room-temperature self-healing but insufficient mechanical properties, in this study, different halloysite-filled PUs were synthesized by using a simple synthesis procedure (the one-shot method), and their self-healing ability and mechanical properties were assessed. In order to allow for a better dispersion of halloysite particles among the PU chains, halloysite was added to the polyol to favor the intercalation of halloysite particles among the soft segments; in previous studies, halloysite was added to isocyanate to facilitate the reaction with hydroxyl groups leading to covalent bonds [40]. It is well known that the addition of fillers causes physical crosslinking with polymer chains [42,43,44,45], which reduces their mobility and, thus, reduces/removes self-healing, so the main objective of this study was to determine the optimal halloysite filler loading that maximizes mechanical reinforcement while preserving the room-temperature self-healing ability of PUs.

## 2. Materials and Methods

### 2.1. Materials

The raw materials used in the synthesis of PUs were 4,4′-methylene bis(cyclohexyl) isocyanate (HMDI) (Sigma Aldrich, St. Louis, MO, USA), 1,4-butanediol (Panreac, Darmstadt, Germany) (chain extender), polycarbonate of 1,6-hexanediol with a molecular weight of 1000 Da (Covestro, Leverkusen, Germany) (polyol), and halloysite (Al_2_Si_2_O_5_(OH)_4_·2H_2_O) filler (Sigma Aldrich, Madrid, Spain).

### 2.2. Methods

#### 2.2.1. Synthesis of the Polyurethanes

Six PUs were synthesized using the one-shot method. Polyol, chain extender, isocyanate, and halloysite were mixed simultaneously. Different weight percentages (0.5, 1, 3, and 10 wt.%) of halloysite were added. An NCO/OH ratio of 1.1 was used and the reactant equivalents were 1.3 × 10^−2^ eq HMDI, 7.8 × 10^−2^ eq polyol, and 8.8 × 10^−4^ eq 1,4-butanediol. The hard segment content of the PUs was 22 wt.%.

The nomenclature of PUs consists in the capital letter “E” followed by a number indicating the weight of added halloysite. For example, E0.5 corresponds to the polyurethane containing 0.5 wt.% halloysite. E0 corresponds to the unfilled polyurethane.

All PUs, except for E0.5-20, were synthesised using halloysite thermally treated at 120 °C overnight to remove absorbed water. E0.5-20 was synthesized using as-received halloysite in order to evaluate the influence of the thermal treatment on the structure and properties of the PUs.

The synthesis procedure of PUs is shown in Figure S1 in the Supplementary Materials. The polyol and 1,4-butanediol were placed in a 60 mL polypropylene bottle and preheated at 80 °C for 10 min. The halloysite was added to the mixture and stirred in a SpeedMixer DAC 150.1 FVZ-K double centrifuge (FlackTek Inc., Landrum, SC, USA) at 2400 rpm for 1 min. Then, HMDI was added, stirred again at 2400 rpm for 1 min, and transferred to an oven for curing through a staged thermal cycle starting at 50 °C and heating at 60 °C, 70 °C, and 80 °C over 8 h. After 24 h at room temperature, the PUs were annealed at 85 °C for 1 h.

#### 2.2.2. Experimental Techniques

**Figure 5:**
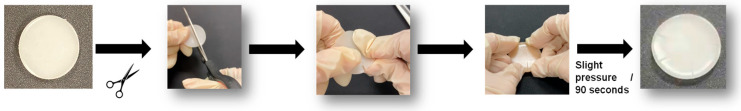
Scheme of the intrinsic self-healing test performed in PU discs. The process involves (from left to right): the initial specimen; partial cutting of the disc; re-joining of the cut pieces; manual pressing for 90 s; and the final healed PU.

The intrinsic self-healing capability of the PUs was evaluated qualitatively at room temperature (20 °C) by using a cutting/re-joining protocol (Figure 5). Circular PU discs were partially sectioned using scissors. Immediately after cutting, both cut surfaces were manually realigned and pressed together under light pressure for 90 s at room temperature. The healed PUs were then subjected to manual tensile stress to qualitatively evaluate mechanical continuity across the re-joined zone (Video S1 in the Supplementary Materials).

The chemical composition of the PUs and halloysite was analyzed by Attenuated Total Reflectance Infrared Spectroscopy (ATR-IR). The ATR-IR spectra were acquired with an Alpha spectrometer (Bruker Optik GmbH, Ettlingen, Germany) equipped with a germanium prism. A total of 60 scans per sample with a resolution of 4 cm^−1^ were carried out.

X-ray photoelectron spectroscopy (XPS) was used to determine the elemental chemical composition on PU–halloysite surfaces. Measurements were performed using a Thermo Scientific NEXSA instrument (Thermo Fisher Scientific, Waltham, MA, USA) provided with a twin crystal monochromator and hemispherical analyzer. A sample spot with a 400 µm diameter was analyzed by using Aluminum kα radiation (1486.6 eV), a current of 3 mA, and a voltage of 12 kV. Survey scans with pass energies of 200 eV were obtained and high-resolution C1s and O1s spectra were obtained by using pass energies of 50 eV.

DSC curves of PUs and halloysite were obtained under a nitrogen atmosphere (flow: 50 mL/min) in a Q100 DSC system (TA Instruments, New Castle, DE, USA). A mass of 5.5–7.1 mg was used and three consecutive scans were carried out: *(i)* heating from −80 °C to 200 °C at a heating rate of 10 °C/min; *(ii)* cooling from 200 °C to −80 °C at a cooling rate of 10 °C/min; and *(iii)* heating from −80 °C to 250 °C at a heating rate of 10 °C/min. Different thermal events were determined from DSC curves of the PUs, mainly the glass transition temperature and the heat capacity at constant pressure (Δc_p_) in the glass transition region; i.e., the endothermal heat involved during the glass transition, and the melting processes.

The crystallinity of PUs and halloysite was analyzed by X-ray diffraction (XRD) using a Bruker D8-Advance diffractometer (Bruker, Ettlingen, Germany). A Kritalloflex K 760-80F X-ray source (3000 W; 20–60 kV; 5–80 mA) and a copper wavenumber of λ = 1.5406 Å were employed. Scanning of 2θ angles from 5° to 90° by increments of 0.05° every 3 s was carried out.

The thermal properties of halloysite and the structural properties of PUs were evaluated in a TGA Q500 instrument (TA Instruments, New Castle, DE, USA). The experiments were conducted under a nitrogen atmosphere (flow: 50 mL/min) to minimize the risk of unwanted oxidation. A total of 9–10 mg of PU was placed in a platinum crucible and heated from 35 to 600 °C by using a heating rate of 10 °C/min.

Scanning electron microscopy (SEM) was used to examine the morphology of halloysite and the dispersion of halloysite particles in the PU matrix. A JEOL IT500HR/LA microscope (Jeol, Tokyo, Japan) equipped with a field emission gun and operating at an accelerating voltage range of 0.5 to 30 kV was used. The samples were coated with platinum for imparting conductivity.

The viscoelastic properties of PUs were evaluated using plate–plate rheology in a DHR-2 rheometer (TA Instruments, New Castle, DE, USA). An upper stainless-steel plate with a diameter of 20 mm was used. The PUs were placed on the lower stainless-steel plate and melted at 120 °C; then, the upper plate was placed on the melted PU and the gap was adjusted to 0.40 mm. The melted PU was cooled down from 120 °C to −20 °C at a controlled cooling rate of 5 °C/min and at a constant frequency of 1 Hz.

Standardized dog-bone PU specimens (ASTM D638-14) [46] were used for stress–strain tests. The tests were carried out in a Zwick/Roell universal testing machine (Barcelona, Spain), and a crosshead speed of 100 mm/min was used.

## 3. Results and Discussion

### 3.1. Characterization of Halloysite

As-received halloysite may contain absorbed water that may affect the extent of its dispersion into the PU matrix. Therefore, before characterization, the halloysite was heated at 120 °C overnight.

**Figure 6:**
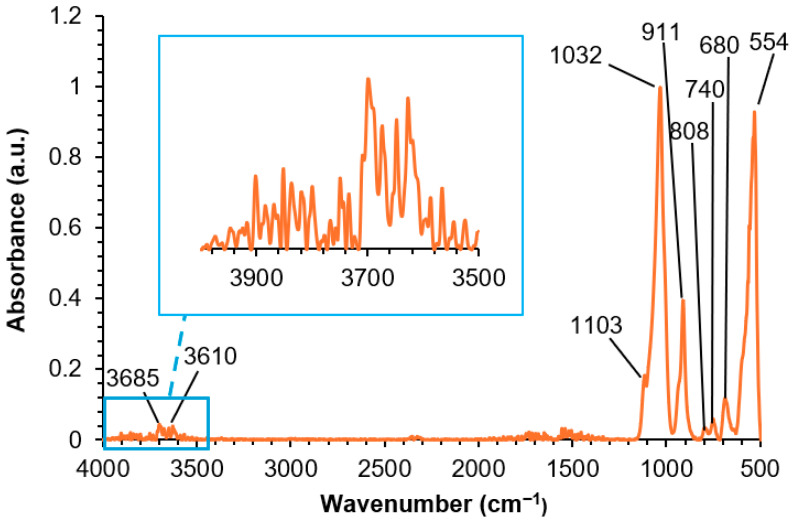
ATR-IR spectrum of halloysite.

The ATR-IR spectrum of halloysite (Figure 6) shows two low and intense broad stretching bands of OH groups at 3685 and 3610 cm^−1^, and Si–O and Si–O–Si stretching bands at 1103 and 1032 cm^−1^, respectively. Si–O bending bands appear at 808 and 740 cm^−1^, and two additional bands at 911 cm^−1^ (Al–OH) and 554 cm^−1^ (Al–O–Si bending) can be distinguished. The assignment of these bands agrees well with the existing literature [34].

The chemical composition on the halloysite surface was assessed by XPS. The halloysite surface contained 71 at.% oxygen, 16 at.% silicon, and 13 at.% aluminium. Thus, the experimental Si/Al ratio was 1.2, which is higher than the stoichiometric Si/Al ratio in halloysite (Al_2_Si_2_O_5_(OH)_4_·nH_2_O), because Si atoms were predominantly located on the outer surfaces of the nanotubes [47]. On the other hand, both the stoichiometric O/Si and O/Al ratios in halloysite are 0.40, while the experimental ratios were 0.26 and 0.18, respectively; therefore, the number of oxygen atoms on the halloysite surface was significantly lower than the stoichiometric one.

The X-ray diffractogram of halloysite (Figure S2 and Table S1 in the Supplementary Materials) shows intense crystalline diffraction peaks at 2θ values of 12°, 20°, 25°, 35°, and 63°, which correspond to the (001), (100), (002), (100), and (300) planes, respectively [40].

**Figure 7:**
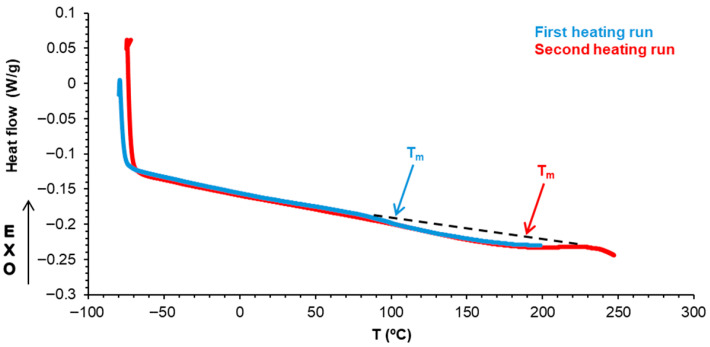
DSC curves of halloysite corresponding to the first and second heating runs.

The thermal properties of halloysite were characterized by DSC and TGA. Figure 7 shows the DSC curves of halloysite corresponding to the first and second heating runs. An endothermic event corresponding to a melting peak at 168 °C with a melting enthalpy of 4.6 J/g was observed. This melting peak corresponds to the onset of the dehydroxylation of inner hydroxyl groups in halloysite [41].

The TGA curve of halloysite shows 15 wt.% loss and exhibits two thermal decompositions at 44 °C (1 wt.% loss due to adsorbed water) and 455 °C (14 wt.% attributed to structural dehydroxylation) (Figure S3 in the Supplementary Materials).

### 3.2. Polyurethanes Made with 0.5 wt.% As-Received and Thermally Treated Halloysite (E0.5-20 and E0.5)

In Section 3.1, it was concluded that the thermal treatment of halloysite at 120 °C caused 1 wt.% water loss and the onset of the dehydroxylation of inner hydroxyl groups. These structural changes may affect the structure and properties of the filled polyurethanes. So, a comparison of the self-healing, structural/morphological, and mechanical properties of two PUs synthesized similarly and containing 0.5 wt.% as-received (E0.5-20) and thermally treated (120 °C/overnight) (E0.5) halloysite was carried out.

**Figure 8:**
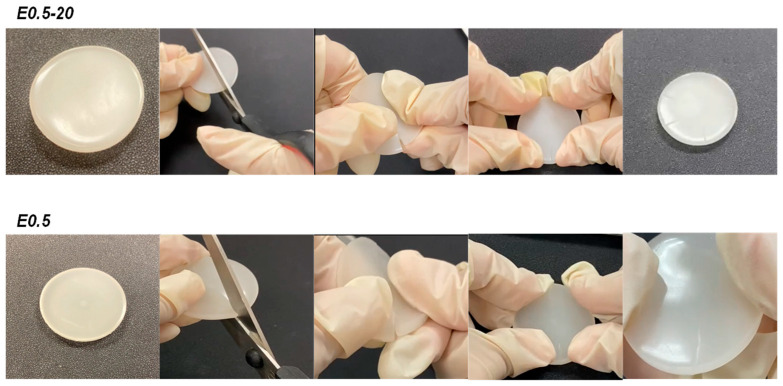
Self-healing test at room temperature of E0.5-20 and E0.5.

Both E0.5-20 and E0.5 exhibit similar and fast intrinsic room-temperature self-healing (Figure 8, Video S1 in the Supplementary Materials), so the addition of as-received and thermally treated halloysite does not affect the self-healing ability of the unfilled polyurethane.

**Figure 9:**
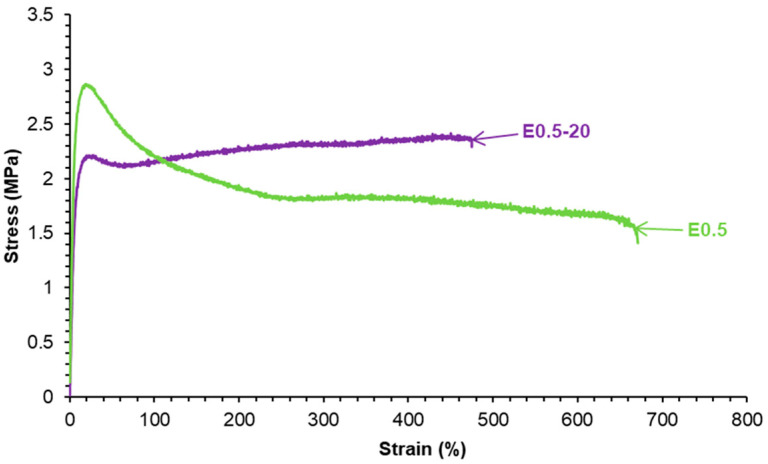
Stress–strain curves of E0.5-20 and E0.5.

The stress–strain curves reveal differences in the mechanical performance of E0.5-20 and E0.5 (Figure 9). Both halloysite-filled PUs exhibit reasonable mechanical properties. E0.5 has a higher Young’s modulus (39.8 MPa), yield point (2.9 MPa), and elongation-at-break value (559%) than E0.5-20 (Young’s modulus, 14.0 MPa; yield point, 2.2 MPa; elongation-at-break value, 396%). However, in the plastic region, E0.5-20 has higher stress and tensile strength (2.3 MPa) than E0.5 (1.6 MPa). The differences in the mechanical properties align with distinct halloysite–PU interactions in E0.5-20 and E0.5.

**Figure 10:**
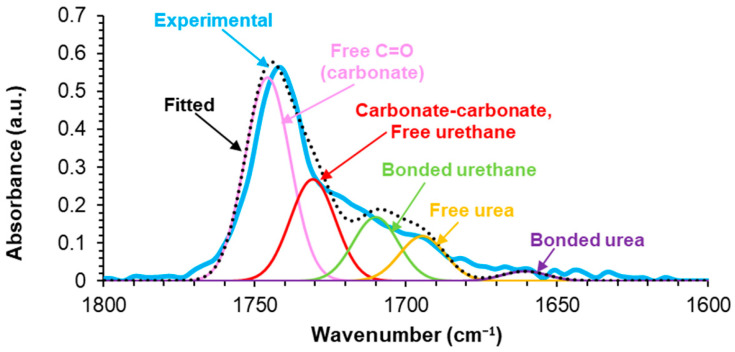
Curve fitting of the carbonyl stretching region of the ATR-IR spectrum of E0.5.

To assess the different mechanical properties between E0.5-20 and E.05, their chemical compositions were analyzed by ATR-IR and XPS. The ATR-IR spectra (Figure S4 in the Supplementary Material file) of E0.5-20 and E0.5 are very similar, but they mainly differ in the carbonyl stretching region (1800–1600 cm^−1^) and the intensity of the OH stretching band of halloysite (more intense in E0.5-20). The curve fitting of the carbonyl stretching region of the ATR-IR spectra of E0.5-20 and E0.5 (Figure 10 and Figure S5 in the Supplementary Materials) was carried out by using a Gaussian function. The C=O stretching regions of the ATR-IR spectra of both PUs exhibit the same contributions due to free carbonate (1745 cm^−1^), free urethane and carbonyl–carbonyl interaction (1730 cm^−1^), hydrogen-bonded urethane (1710 cm^−1^), free urea (1695 cm^−1^), and hydrogen-bonded urea (1660 cm^−1^) groups. This assignment was made according to a previous study [12]. Both PUs show similar percentages of bonded urethane (14–15%), free urea (10–11%), and bonded urea (2%), indicating that the addition of as-received or thermally treated halloysite does not affect the formation of free and hydrogen-bonded urea (Table 1). However, E0.5 has a higher percentage of free carbonate groups and a lower percentage of free urethane/carbonate–carbonate interactions than E0.5-20 (Table 1). Because the hard segment contents in E0.5-20 and E0.5 are similar (22 wt.%), the differences in the percentages of free and associated carbonate groups are due to lower interactions between the carbonate groups in E0.5, leading to the higher mobility of the polyurethane chains.

The structural differences between E0.5-20 and E0.5 were confirmed by DSC. The DSC curves of both PUs (Figure S6 in the Supplementary Materials) show the same thermal events (the glass transition of the soft segments at −20–−23 °C, the melting of the soft segments at 42 °C, and one small melting event at 91–100 °C) (Table 2). E0.5 shows a significantly lower (0.20 J/g °C) heat capacity at constant pressure (Δc_p_) in the glass transition region and significantly higher melting enthalpy (7.5 J/g) due to the higher amount of free carbonate groups and, thus, the higher mobility of the soft segments. E0.5-20 shows a Δc_p_ value of 0.67 J/g °C and a melting enthalpy of 2.0 J/g, which correspond to a lower number of free carbonate groups and lower mobility of the soft segments (Table 2). Therefore, E0.5 shows a more ordered internal structure and stronger interactions between soft segments—likely reinforced by more effective halloysite–PU interfacial interactions—than E0.5-20. On the other hand, the small melting event at 91–100 °C in the DSC curves of both PUs is likely a partial relaxation or reorganization of the polyurethane chains around the halloysite surface because the melting in the DSC curve of halloysite (Figure 7) is produced at a higher temperature. It should be noted that the glass transition temperatures of E0.5 and E0.5-20 cannot be obtained due to the overlap with the end of the melting peak due to the onset of the dehydroxylation of inner hydroxyl groups in halloysite (Figure 7).

**Figure 11:**
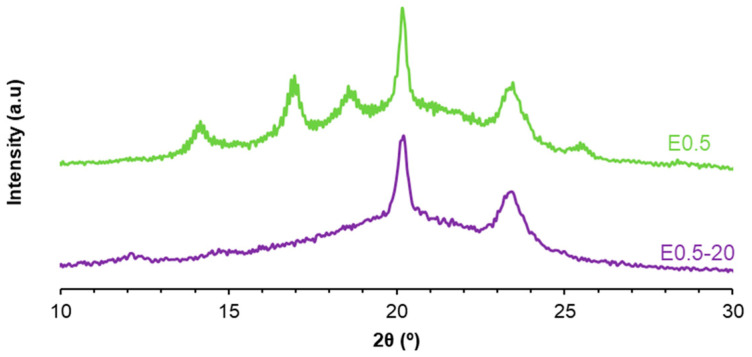
X-ray diffractograms of E0.5 and E0.5-20.

Because the DSC curves show a different ordering of the soft segments in E0.5-20 and E0.5, they should exhibit different crystalline structures. The X-ray diffractogram of E0.5-20 shows a higher number of peaks than E0.5 and exhibits more intense peaks at 2θ = 20° (3018 a.u. vs. 1918 a.u.) and 2θ = 23° (1918 a.u. vs. 352 a.u.) (Figure 11), indicating higher crystalline ordering of the soft segments. On the other hand, the intense halloysite peak at 2θ = 12° appears only in E0.5-20, whereas the X-ray diffractogram of E0.5 shows the peaks of halloysite at 2θ values of 17°, 19°, and 25°. Therefore, the halloysite–PU interactions are different in E0.5-20 and E0.5.

**Figure 12:**
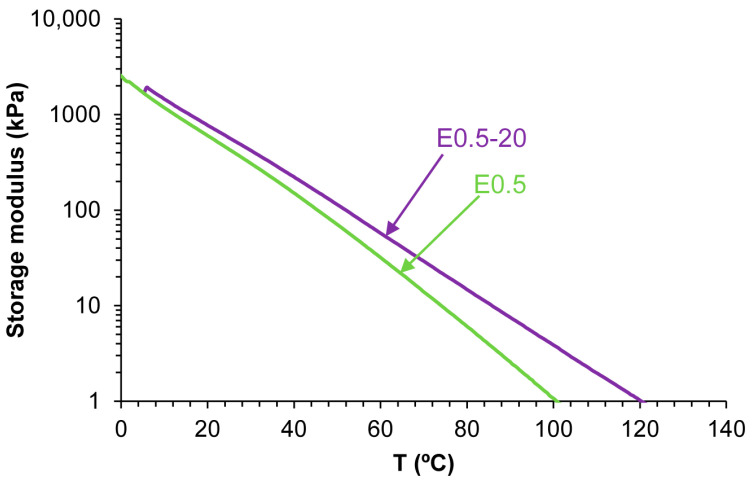
Variation in the storage modulus as a function of the temperature for E0.5 and E0.5-20.

The different structures of E0.5-20 and E0.5 also determine their viscoelastic properties. Figure 12 shows the variation in the storage modulus (G′) as a function of the temperature for E0.5 and E0.5-20. Both PUs show a continuous decrease in the G′ value by increasing the temperature, and the G′ values are higher in E0.5-20 in all temperature ranges. Therefore, E0.5-20 shows more elastic rheological behavior than E0.5 due to stronger halloysite–polyurethane chain interactions that may constrain the mobility of polymer chains.

**Figure 13:**
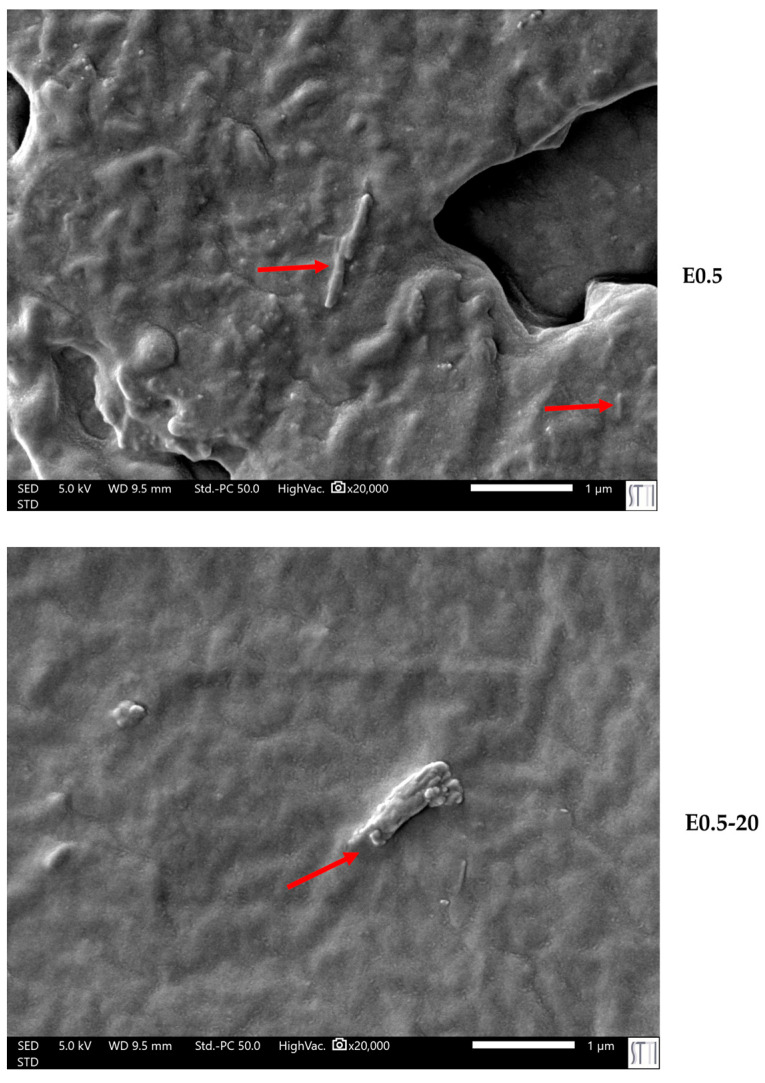
SEM micrographs of E0.5 and E0.5-20.

The differences in the viscoelastic behavior are closely related to the microstructural morphology of the polyurethanes. SEM micrographs (Figure 13) reveal differences in the dispersion and morphology of halloysite particles within the matrix. While E0.5 exhibits a homogeneous dispersion of tubular halloysite particles embedded within the polymer network, E0.5-20 presents less well-dispersed halloysite aggregates, which tend to form small clusters. This structural disparity supports the different chain mobilities and viscoelastic responses in E0.5-20 and E0.5.

The different mechanical properties and structures of E0.5-20 and E0.5 could be ascribed to the different reactivities of water and hydroxyl groups of halloysite with isocyanate. Because the as-received halloysite contains two molecules of water and surface hydroxyl groups, urea bonds may form by reaction with isocyanate. Thermally treated halloysite does not contain significant amounts of water and has a lower number of hydroxyl groups than as-received halloysite, so the formation of urea bonds should be inhibited. However, the curve fitting of the ATR-IR spectra of E0.5-20 and E0.5 shows similar numbers of urea groups, indicating similar reactivities of halloysite with isocyanate in both PUs. Considering that, during PU synthesis, halloysite was initially dispersed between the polyol chains, it is quite unlikely that a reaction of halloysite with isocyanate can be produced (the number of OH groups is significantly higher in the polyol than in halloysite), so the differences between E0.5-20 and E0.5 derive from the different interactions of the as-received and thermally treated halloysite with the soft segments. This is supported by the higher percentage of free carbonate groups, the higher heat capacity at constant pressure (Δc_p_) in the glass transition region, the lower crystallinity, and the lower rheological elastic modulus in E0.5.

### 3.3. Characterization of Polyurethanes Without and with Different Amounts of Halloysite

The experimental evidence shown in Section 3.2 indicated that the thermally treated halloysite is better dispersed in PU than the as-received one, so different amounts (0.5 to 10 wt.%) of thermally treated halloysite were added during PU synthesis to determine the optimal halloysite content that offers the best balance between mechanical reinforcement and room-temperature intrinsic self-healing.

**Figure 14:**
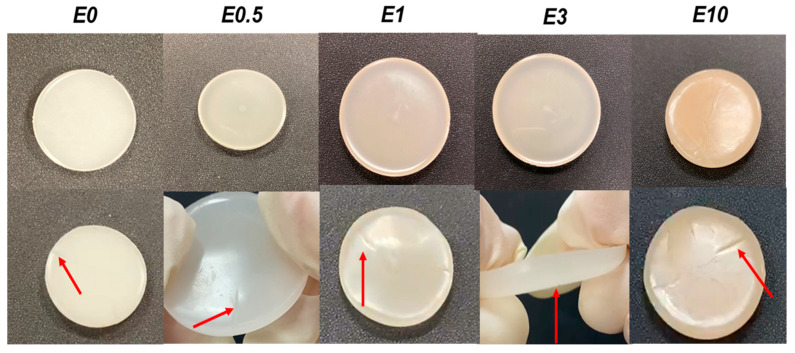
Visual aspect of unfilled and halloysite-filled polyurethanes before (top row) and after (bottom row) the intrinsic room-temperature self-healing test. Red arrows indicate the location of the initial cut.

All PUs exhibited fast intrinsic self-healing at room temperature (90 s under mild pressure) (Figure 14). E0 (without halloysite) exhibited self-healing due to the segmental mobility of the polycarbonate soft segments [12]. Similarly, all halloysite-filled PUs (E0.5, E1, E3, and E10) exhibited intrinsic self-healing at room temperature, indicating that the addition of halloysite does not inhibit the dynamic non-covalent interactions between carbonate groups in E0 (Figure 2), even by adding 10 wt.% halloysite.

**Figure 15:**
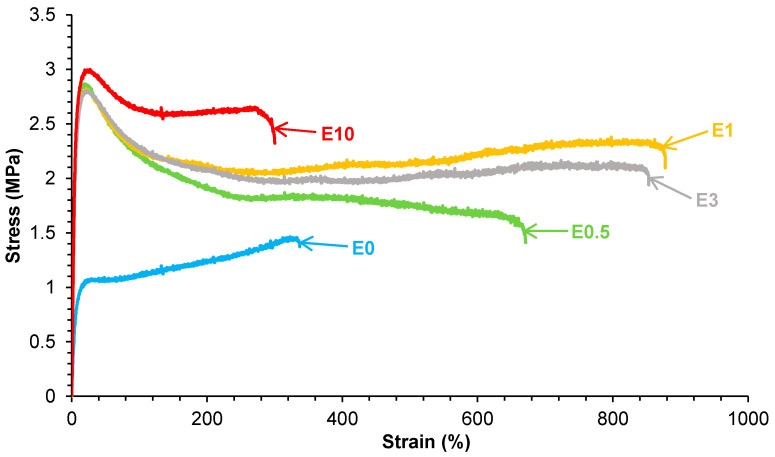
Stress–strain curves of the polyurethanes without and with different numbers of HNTs.

The stress–strain curves (Figure 15) show that all PUs containing halloysite have significantly improved mechanical properties with respect to E0 (Table 3). The Young’s modulus, yield strength, and elongation-at-break values increased noticeably by adding 0.5 wt.% halloysite only, and E0.5 also exhibited room-temperature self-healing. In general, Young’s moduli and yield point values did not change noticeably by increasing the halloysite loading in the PUs, but the tensile strength and elongation-at-break values were higher in E1 and E3. However, the addition of 10 wt.% halloysite caused a stiffening of the polyurethane, i.e., higher tensile strength and lower elongation-at-break values than in E3 were obtained. This suggests that the addition of an excessive amount of halloysite filler leads to particle aggregation and reduced matrix flexibility, offsetting the reinforcement benefits, in agreement with previous studies [40,41,42,43,44]. This can be ascribed to the higher elastic modulus of halloysite than the one of E0 and strong halloysite–PU interfacial interactions, which limited the motion of polyurethane chains. The mechanical reinforcement of PUs by adding halloysite did not compromise their intrinsic room-temperature self-healing ability. However, even though they improved with respect to E0, the values of the mechanical parameters of the fast room-temperature self-healing halloysite-filled PUs were lower than the ones of the thermally self-healing halloysite-filled PUs [42,43,44].

The influence of adding different amounts of halloysite on the chemical structure of the PUs was determined by ATR-IR spectroscopy and XPS.

The ATR-IR spectra of PUs without and with different amounts of halloysite (Figure S7 in the Supplementary Materials) show a broad band at 3351 cm^−1^ (N–H stretching of urethane + O–H stretching of halloysite) that becomes more intense and broader by increasing the halloysite content. Furthermore, the intensity of the Al–O–Si band at 554 cm^−1^ increases by increasing the halloysite content in the PUs. The bands at 2928 and 2847 cm^−1^ are due to the symmetric and asymmetric stretching of –CH_2_ groups and the bands at 1400 and 1457 cm^−1^ correspond to –CH_2_ groups of the soft segments and the chain extender. These bands are slightly less intense in the PUs containing higher amounts of halloysite. On the other hand, the C-O stretching bands of the soft segments at 789, 975, 1020, and 1049 cm^−1^ show higher intensities in E3 and E10, indicating the interaction of halloysite with the soft segments.

All ATR-IR spectra show an intense C=O stretching band at 1741 cm^−1^ due to carbonate and urethane groups and a band of N–H bending + C–N stretching at 1534 cm^−1^ of urethane groups. The curve fitting of the carbonyl region (1800–1600 cm^−1^) of the PUs shows similar C=O species (Figure S8 in the Supplementary Materials), but, depending on their halloysite content, they differ in their percentages. Table 4 shows that the percentages of hydrogen-bonded urethane at 1710 cm^−1^ (14–15%), free urea at 1695 cm^−1^ (9–11%), and hydrogen-bonded urea at 1660 cm^−1^ (2–4%) are similar in unfilled and filled PUs, and they remain unchanged with the addition of different amounts of halloysite. On the other hand, the addition of 0.5 wt.% halloysite does not affect the percentages of free carbonate at 1745 cm^−1^ (48–49%) and carbonate–carbonate interactions at 1730 cm^−1^ (24–25%) in E0. However, a decrease in the percentage of free carbonate from 49% in E0 to 39% and an increase in the percentage of carbonate–carbonate interactions from 25% in E0 to 33% are observed in E1 and E3, indicating the disruption of carbonate–carbonate interactions between the soft segments by halloysite addition, i.e., new halloysite–PU interactions are produced by constrained polymer regions near the filler interface. Comparable percentages of free carbonate and carbonate–carbonate interactions in E3 and E10 indicate that the addition of more than 3 wt.% halloysite does not further alter the chemical structure of E0.

The surface chemical composition of selected PUs was further examined by XPS. The presence of halloysite was confirmed by the detection of 0.5 and 1 at.% Si on E0.5 and E1 surfaces, respectively (Table 5). The atomic percentages of carbon and oxygen are slightly different on E0.5 and E1 surfaces. The E0.5 surface exhibits a lower carbon and a higher oxygen content compared with the E1 surface. This trend correlates with the higher free carbonate group content on the E0.5 surface with respect to the E1 surface (in agreement with the ATR-IR spectra), suggesting a higher proportion of accessible oxygen-rich species on the E0.5 surface.

These differences are also reflected in the high-resolution C1s spectra of the halloysite-filled PUs (Figure S9 in the Supplementary Materials). The chemical species on E0 and E1 surfaces are similar: 87 at.% C-C/C-H (binding energy, BE = 284.7 eV), 9 at.% C–N due to urethane (BE = 286.4 eV), 3 at.% C=O due to urethane and carbonate (BE = 288.6 eV), and 1 at.% O–(C=O)–O due to carbonate (BE = 290.4 eV) (Table 6). Therefore, the addition of 1 wt.% halloysite does not significantly change the chemical species on the E1 surface, i.e., the carbonate–carbonate interactions are not affected by adding filler. However, the addition of 0.5 wt.% halloysite increases the percentages of C–N and O–(C=O)–O species and decreases the percentage of C=O species with respect to the E0 surface (Table 6). The increase in O–(C=O)–O content, combined with the reduction in the C=O percentage, implies a greater presence of free carbonate groups and a partial disruption of carbonate–carbonate interactions on the E0.5 surface, possibly caused by the interaction of halloysite with the soft segments.

Similar findings are evidenced in the high-resolution O1s XPS spectra of the halloysite-filled PUs (Figure S10 in the Supplementary Materials). All PUs show C=O (531.6–531.8 eV) and C–O (533.5–533.7 eV) species. The addition of 0.5 wt.% halloysite led to a decrease (from 75% to 67%) in C=O and an increase (from 25% to 33%) in C–O species on the E0.5 surface compared with the E0 surface (Table 7), suggesting a higher content of free carbonate groups and partial disruption of carbonate–carbonate interactions due to halloysite interactions with the soft segments. By contrast, on the E1 surface, the percentages of C=O and C–O species remained nearly unchanged relative to the E0 surface, indicating that the addition of 1 wt.% halloysite does not significantly affect the carbonate interactions among the soft segments.

Halloysite interactions affect the thermal properties of PUs. The DSC curve of the unfilled PU—E0—only shows the glass transitions of the soft segments at −21 °C and the hard segments at 223 °C (Figure S11 in the Supplemental Materials). The addition of any amount of halloysite does not change the glass transition temperature (T_g_) of the soft segments, but the heat capacity at constant pressure (∆c_p_) values are higher in E1 and E3 (0.32–0.37 J/g. °C) (Table 8), indicating the confinement of the soft segments between halloysite particles. Furthermore, two new melting transitions at 39–42 °C and 61–100 °C appear in filled PUs, indicating the existence of halloysite–PU interactions (Table 8). The thermal event at 39–42 °C is due to the melting of halloysite–soft segment interactions and the melting enthalpy is significantly higher in E0.5, indicating more effective intercalation of halloysite particles among the soft segments of E0. The melting at 61–100 °C is associated with halloysite (the DSC curve of halloysite shows a melting event at 168 °C with an enthalpy of 4.6 J/g, Figure 7) and, in general, the melting temperature and enthalpy are lower in the filled PUs. This evidence supports the intercalation of halloysite particles among the soft segments.

The addition of halloysite increases the thermal stability of the PUs (Figure S12 in the Supplementary Materials) in a similar manner irrespective of the amount added. Furthermore, the TGA curves of halloysite-filled PUs shift toward higher temperatures compared with the unfilled PU (E0), indicating the existence of halloysite–PU interactions. The better thermal stability of halloysite-filled PUs has been previously stated in thermally self-healing PUs [43]. However, the increase in thermal stability by adding 0.5–10 wt.% halloysite does not agree with a previous study dealing with thermal self-healing PUs in which the weight loss was shifted to lower temperatures for 0.5–1 wt.% halloysite [45]; conversely, increased thermal stability was found by adding more than 1 wt.% halloysite, in agreement with the results of this study. This discrepancy is caused by the different synthesis procedures, the addition of halloysite to the polyol, and the composition of PUs.

**Figure 16:**
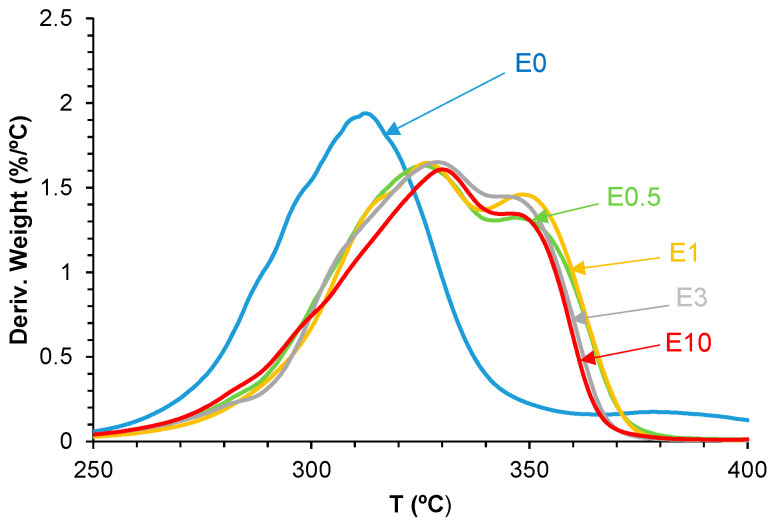
Derivative of TGA curves of the polyurethanes without and with different amounts of halloysite.

As shown in Figure 16, all filled PUs exhibit an additional thermal degradation step at 324–330 °C with increasing mass losses by increasing the halloysite loading, attributed to new interactions between halloysite and PU. Furthermore, the hard segments decompose at lower temperatures (281–283 °C) and with lower mass loss (7%) in all PUs containing halloysite with respect to E0, indicating the intercalation of halloysite among the polyurethane chains that facilitate earlier decomposition (Table 9). Also, the degradation of the soft segments at 313 °C is gradually displaced to lower temperatures and with lower mass loss by increasing the halloysite content in the PUs. Additionally, the thermal degradation of the carbonate–carbonate interactions at 387 °C in E0 is displaced to lower temperatures (346–348 °C) with mass losses of 27–33% in all filled PUs because of the intercalation of halloysite nanoparticles among the polyurethane chains.

**Figure 17:**
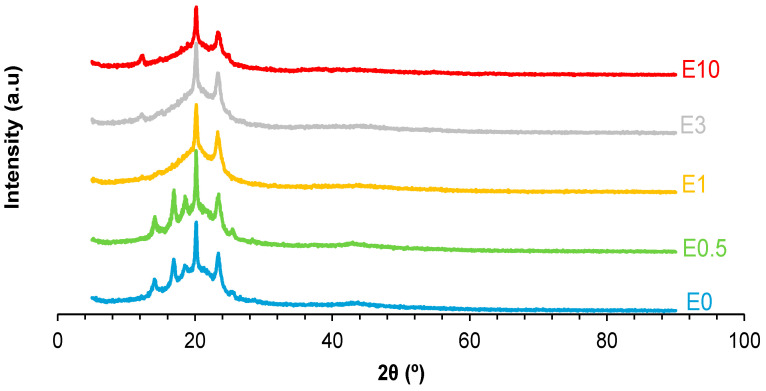
X-ray diffractograms of polyurethanes without and with different amounts of halloysite.

The intercalation of halloysite particles among polymer chains should affect the crystallinity of PUs. The X-ray diffractogram of E0 (Figure 17) shows several peaks at 2θ values of 14°, 17°, 19°, 20°, 23°, 25°, and 44° (Table S2 in the Supplementary Materials), and the most intense peak is the one at 2θ = 20°. The X-ray diffractogram of E0.5 closely resembles that of the unfilled PU (E0), but with lower intensities of the peaks, except for the ones at 2θ values of 19° and 25°. Since these diffraction peaks are absent in the X-ray diffractogram of neat halloysite, their enhancement in E0.5 suggests deep intercalation of halloysite within the PU chains, disrupting the crystallite packing of the soft segments. In contrast, the X-ray diffractograms of E1, E3, and E10 do not exhibit the peaks at 2θ values of 17°, 19°, and 25°, indicating a reduced degree of intercalation and, potentially, filler aggregation. In fact, in E3 and E10, a peak at 2θ = 12°—characteristic of halloysite—is clearly visible, supporting the presence of halloysite agglomerates in the PUs at higher filler contents. Thus, low halloysite loadings (e.g., 0.5 wt.%) promote better dispersion and interfacial interactions with polymeric chains, while higher contents lead to reduced structural integration.

**Figure 18:**
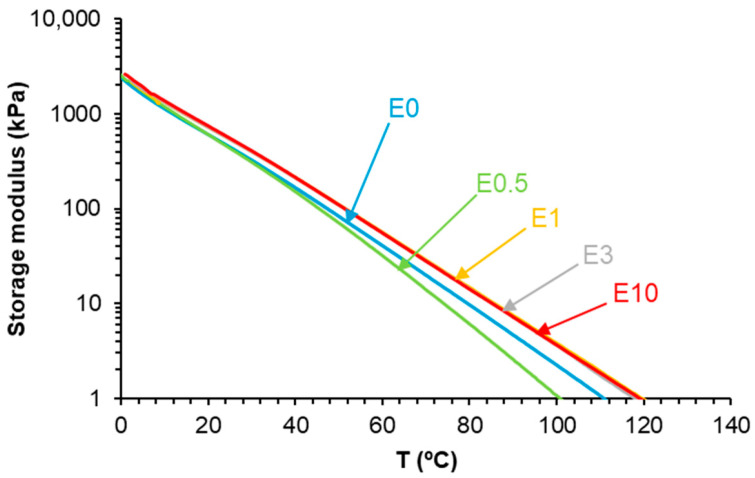
Variation in the storage modulus as a function of temperature for polyurethanes without and with different numbers of HNTs.

The viscoelastic properties of PUs are affected by the existence of halloysite–PU interactions. The rheological behavior of E0.5 differs notably from that of the other filled PUs. As shown in Figure 18, all PUs exhibit a gradual decrease in the storage modulus (G′) by increasing temperature. E0.5 presents lower G′ values than E0 across most of the temperature range, indicating a more mobile network. Despite this, at 5 °C, E0.5 displays a higher G′ (1732 kPa) than E0 (1571 kPa), suggesting that the halloysite intercalation within the soft segments locally reinforces the structure while simultaneously disrupting carbonate–carbonate interactions. In contrast, the rheological curves of E1, E3, and E10 are similar and show consistently higher G′ values than E0, indicating that, at higher halloysite loadings, partial aggregation of halloysite particles is produced. These interactions may compromise uniform dispersion and polymer chain mobility.

**Figure 19:**
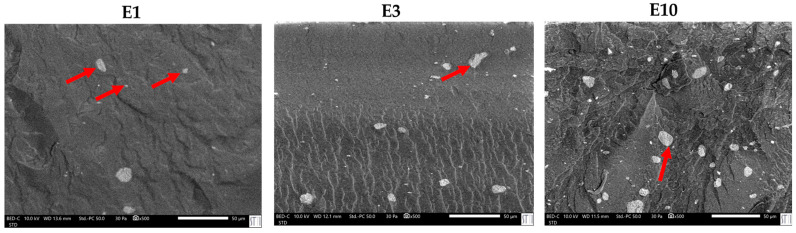
SEM micrographs of the polyurethanes without and with different amounts of halloysite. ×500.

**Figure 20:**
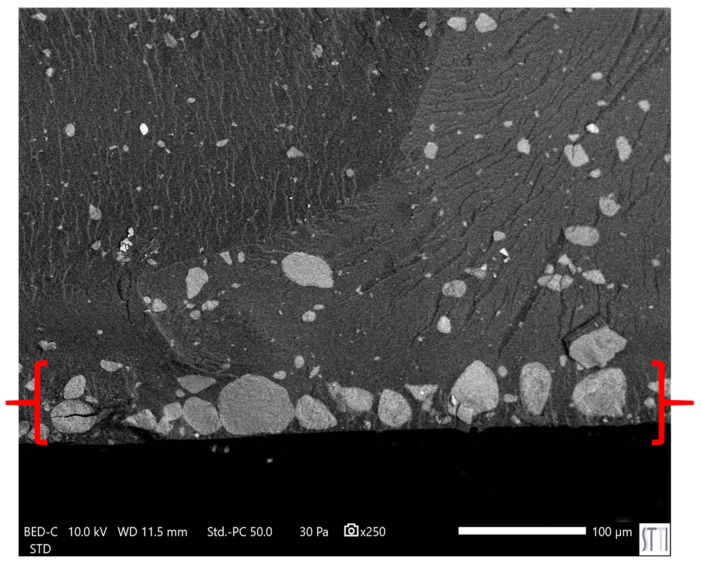
SEM micrograph of E10’s surface. ×250.

In line with the rheological behavior, SEM micrographs of PUs (Figure 19) confirm a distinct dispersion pattern in E0.5 in which well-dispersed nanotubular structures and small individual halloysite particles are embedded uniformly within the polymer matrix. As the halloysite content increases, particle agglomeration becomes more evident. In E1, spherical agglomerates appear, consistent with its broader particle size distribution (0.4–1.3 µm) as compared with E0.5 (Figure S13 in the Supplementary Materials). This trend intensifies in E3 and E10, where larger clusters ranging from 3 to 7 µm are observed, and a significant fraction of halloysite particles appear to have accumulated at the PU surface, suggesting poor filler integration. In particular, E10 shows widespread surface migration and the highest degree of aggregation (Figure 20), which correlates with the previously discussed reduction in polymer–filler interfacial quality and mechanical uniformity at high halloysite loadings. On the other hand, the surface of filled PUs becomes much rougher due to the strong bonding between the halloysite and the polymer matrix, which constrains the polyurethane chains surrounding the filler and increases the mechanical interlocking and physical entanglement density in the matrix; thus, the existence of halloysite favors energy dissipation during fracture.

**Figure 21:**
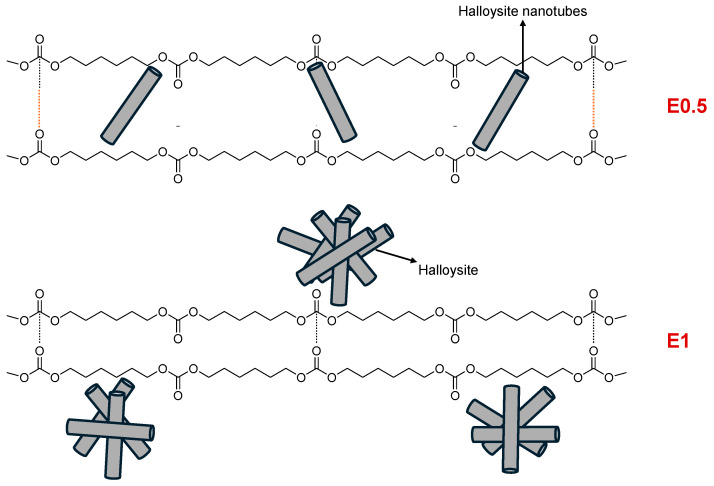
Schematic representation of the interactions between halloysite and soft segments in polyurethanes with different halloysite contents.

The collective results from the thermal, mechanical, structural, and surface analyses suggest that halloysite interacts with the polyurethane matrix via two distinct mechanisms, depending on its concentration. As illustrated in Figure 21, at low content (E0.5), halloysite nanotubes are sufficiently dispersed and capable of intercalating between the polycarbonate soft segments, locally disrupting carbonate–carbonate interactions and reinforcing the matrix. In contrast, at higher concentrations (E1 and PUs with 3–10 wt.% halloysite), halloysite predominantly interacts externally along the polymer chains, leading to more extensive but superficial contact, which limits its effectiveness as a reinforcing agent and disrupts the uniform phase morphology.

## 4. Conclusions

This work demonstrated the successful development of intrinsically self-healing polyurethanes reinforced with thermally treated halloysite, capable of maintaining self-repair at room temperature while achieving notable improvements in thermal and mechanical performance. The experimental results confirmed that thermal treatment of halloysite at 120 °C overnight promoted partial dehydration without compromising its tubular structure, thereby enhancing its compatibility with the polyurethane matrix and minimizing undesired reactions during synthesis.

Among the six PUs, the one containing 0.5 wt.% thermally treated HNTs (E0.5) exhibited the most balanced performance. Compared with its counterpart made with as-received halloysite (E0.5-20), E0.5 showed superior dispersion of nanotubes, greater elongation-at-break values, and higher yield strength. These improvements were associated with a greater proportion of free carbonate groups and enhanced segmental mobility, as confirmed by ATR-IR, DSC, and rheological analysis. Furthermore, SEM micrographs revealed well-dispersed halloysite nanotubes in E0.5, contrasting with the small agglomerates observed in E0.5-20.

All halloysite-filled PUs maintained their intrinsic self-healing ability at room temperature regardless of filler content. Notably, formulations containing 1 and 3 wt.% halloysite (E1 and E3) achieved increased tensile strength, though this was accompanied by the appearance of larger agglomerates and reduced polymer–filler intercalation. The PU with 10 wt.% halloysite (E10) exhibited the least effective dispersion, with extensive aggregation and particle migration toward the surface, leading to diminished mechanical benefits and reduced ductility.

Therefore, the PU with 0.5 wt.% thermally treated halloysite (E0.5) represented the optimal compromise between mechanical reinforcement and room-temperature self-healing performance. These findings highlight the critical role of filler content and dispersion in tuning the functional response of self-healing PUs and underscore the potential of halloysite as a nanostructured additive for the next generation of adaptive PUs.

## Figures and Tables

**Table 1:** Percentages of different C=O species of E0.5-20 and E0.5. Curve fitting of the carbonyl stretching region of the ATR-IR spectra.

Wavenumber (cm^−1^)	Percentage (%)		Assignment
	E0.5-20	E0.5	
1745	41	48	Free C=O (carbonate)
1730	33	24	Carbonate–carbonate Free urethane
1710	14	15	Bonded urethane
1695	10	11	Free urea
1660	2	2	Bonded urea

**Table 2:** Thermal events obtained from the first heating run of DSC curves of E0.5 and E0.5-20.

PU	T_g_ (°C)	Δc_p_ (J/g·°C)	T_m1_ (°C)	ΔH_m1_ (J/g)	T_m2_ (°C)	ΔH_m2_ (J/g)
E0.5-20	−23	0.67	42	2.0	91	0.19
E0.5	−20	0.20	42	7.5	100	0.12

**Table 3:** Some parameters obtained from the stress–strain curves of the polyurethanes without and with different amounts of halloysite.

PU	Young Modulus (MPa)	Yield Stress (MPa)	Tensile Strength (MPa)	Elongation-at-Break (%)
E0	5.2	1.0	1.4	281
E0.5	39.8	2.9	1.4	559
E1	38.5	2.8	2.1	731
E3	44.5	2.8	1.9	711
E10	41.7	3.0	2.3	250

**Table 4:** Percentages of C=O species of polyurethanes without and with different amounts of halloysite. Curve fitting of the carbonyl region (1600–1800 cm^−1^) of the ATR-IR spectra.

Wavenumber (cm^−1^)	Percentage (%)					Assignment
	E0	E0.5	E1	E3	E10	
1745	49	48	42	39	39	Free C=O (carbonate)
1730	25	24	31	33	33	Carbonate–carbonate Free urethane
1710	14	15	14	14	15	Bonded urethane
1695	9	11	11	10	11	Free urea
1660	3	2	2	4	2	Bonded urea

**Table 5:** Chemical composition on the E0, E0.5, and E1 surfaces. Survey XPS.

Element	Percentage (at. %)		
	E0	E0.5	E1
C	84	81	84
O	16	18.5	15
Si		0.5	1

**Table 6:** Binding energies and percentages of carbon species on the E0, E0.5, and E1 surfaces. High-resolution C1s XPS spectra.

Species	Binding Energy (eV)	Percentage (at.%)		
		E0	E0.5	E1
C–C	284.7	87	85	87
C–N	286.4	9	10	9
C=O	288.6	3	2	3
O–(C=O)–O	290.4	1	3	1

**Table 7:** Binding energies and percentages of oxygen species on the E0, E0.5, and E1 surfaces. High-resolution O1s XPS spectra.

Species	Binding Energy (eV)	Percentage (at.%)		
		E0	E0.5	E1
C=O	531.7–531.8	75	67	77
C–O	533.5–533.7	25	33	23

**Table 8:** Thermal events obtained from the first heating run of the DSC curves of the polyurethanes without and with different amounts of halloysite.

PU	T_g_ (°C)	Δ_Cp_ (J/g·°C)	T_m1_ (°C)	ΔH_m1_ (J/g)	T_m2_ (°C)	ΔH_m2_ (J/g)
E0	−21	0.22	-	-	-	-
E0.5	−20	0.20	42	7.49	100	0.12
E1	−22	0.32	39	0.98	76	0.25
E3	−21	0.37	-	-	91	0.98
E10	−22	0.22	41	3.30	61	0.22
Halloysite	-	-	-	-	77	0.66

**Table 9:** Temperatures and weight losses of the thermal degradations for polyurethanes without and with different amounts of halloysite.

PU	1st Degradation		2nd Degradation		3rd Degradation		4th Degradation	
	T_1_ (°C)	Weight Loss_1_ (%)	T_2_ (°C)	Weight Loss_2_ (%)	T_3_ (°C)	Weight Loss_3_ (%)	T_4_ (°C)	Weight Loss_4_ (%)
E0	297	31	313	60	-	-	387	6
E0.5	283	7	312	28	324	32	347	32
E1	-	-	314	33	326	34	348	33
E3	281	7	308	18	328	42	346	29
E10	280	7	294	9	330	51	346	27

## Data Availability

The original contributions presented in this study are included in the article/Supplementary Materials. Further inquiries can be directed to the corresponding author.

## Supplementary Materials

The following supporting information can be downloaded at: https://www.mdpi.com/article/10.3390/polym17202807/s1, Figure S1: Flow diagram of the one-shot synthesis protocol of polyurethanes without and with different amounts of HNTs; Figure S2: X-ray diffractogram of halloysite; Figure S3: (a) TGA curve, and (b) derivative of TGA curve of halloysite; Figure S4: ATR-IR spectra of E0.5 and E0.5-20; Figure S5: Curve fitting of the carbonyl stretching region of the ATR-IR spectrum of E0.5-20; Figure S6: DSC curves of E0.5-20 and E0.5. First heating run; Figure S7: ATR-IR spectra of polyurethanes without and with different amounts of halloysite; Figure S8: Curve fitting of the carbonyl stretching region of the ATR-IR spectrum of E10; Figure S9: High-resolution C1s XPS spectrum of E0.5 surface; Figure S10: High-resolution O1s XPS spectrum of E0.5 surface; Figure S11: DSC curves of polyurethanes without and with different amounts of halloysite. First heating run; Figure S12: TGA curves of polyurethanes without and with different amounts of halloysite; Figure S13: Halloysite particle size distributions in E0.5 and E1; Table S1: 2θ values, intensities and (hkl) planes of the peaks of the X-ray diffractogram of halloysite; Table S2: 2θ values and intensities of the main diffraction peaks of the polyurethanes without and with different amounts of HNTs; Video S1: Room temperature self-healing of E0.5.
