# Supplementary material for: Room-Temperature Self-Healing Polyurethanes Containing Halloysite Clay with Enhanced Mechanical Properties
Source: Polymers (Basel). 2025 Oct 21;17(20):2807. doi: 10.3390/polym17202807 (PMC12567213; doi:10.3390/polym17202807)
Supplement: Supplementary file 1 [file polymers-17-02807-s001.zip › Suppementary Figures and Tables.pdf]

Room-Temperature Self-Healing Polyurethanes Containing Halloysite Clay with Enhanced Mechanical Properties

Eva Dauder-Bosch and José Miguel Martín-Martínez

Synthesis of the polyurethanes

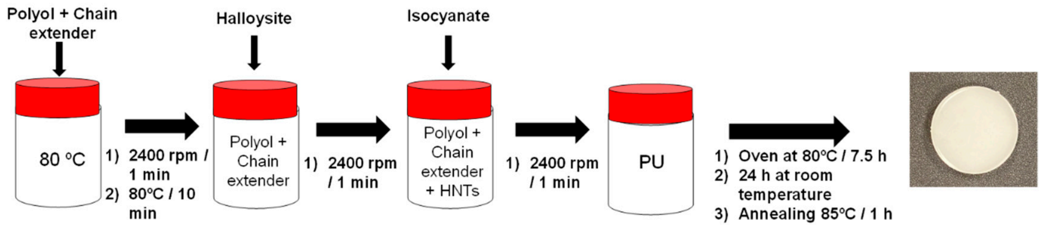

Figure S1. Flow diagram of the one-shot synthesis protocol of polyurethanes without and with different amounts of HNTs.

Characterization of halloysite

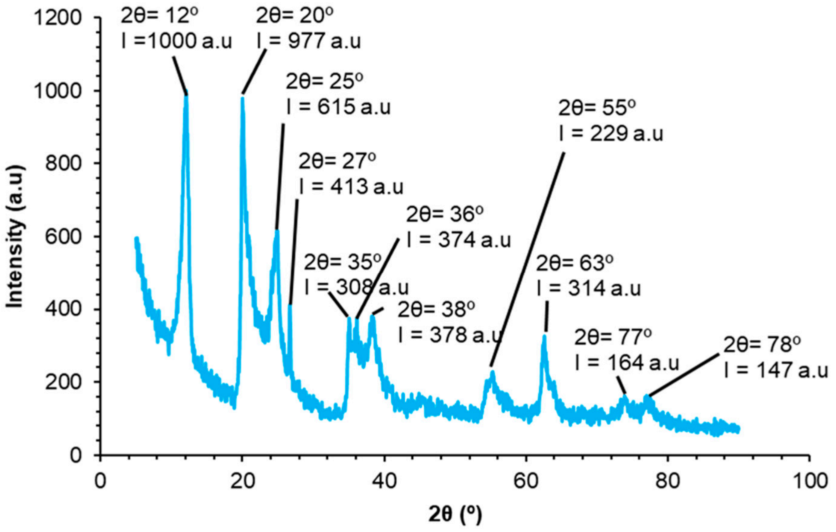

Figure S2. X-ray diffractogram of halloysite.

Table S2. 2θ values, intensities and (hkl) planes of the peaks of the X-ray diffractogram of halloysite.

2θ (°)	Intensity (a.u.)	(hkl) plane
12	1000	(001)
20	977	(100)
25	615	(002)
27	413	
35	308	(100)
36	374	
38	378	(003)
55	229	(210)
63	314	(300)
77	164	
78	147	

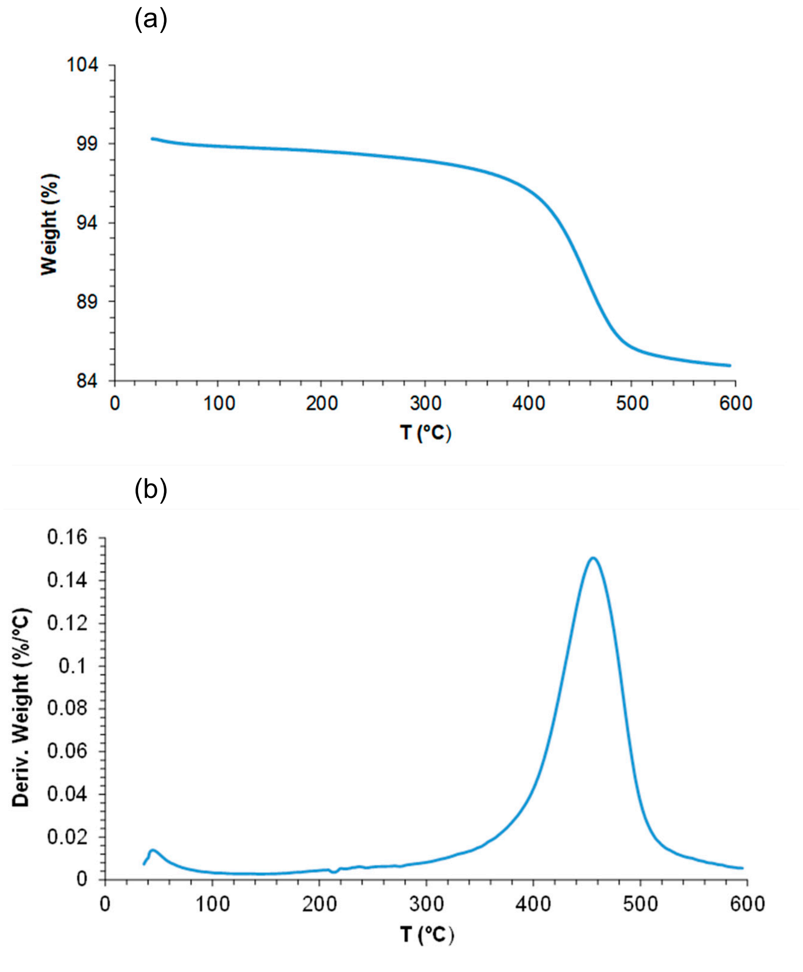

Figure S3. (a) TGA curve, and (b) derivative of TGA curve of halloysite.

Polyurethanes made with 0.5 wt.% as-received and thermally treated halloysite (E0.5-20 and E0.5)

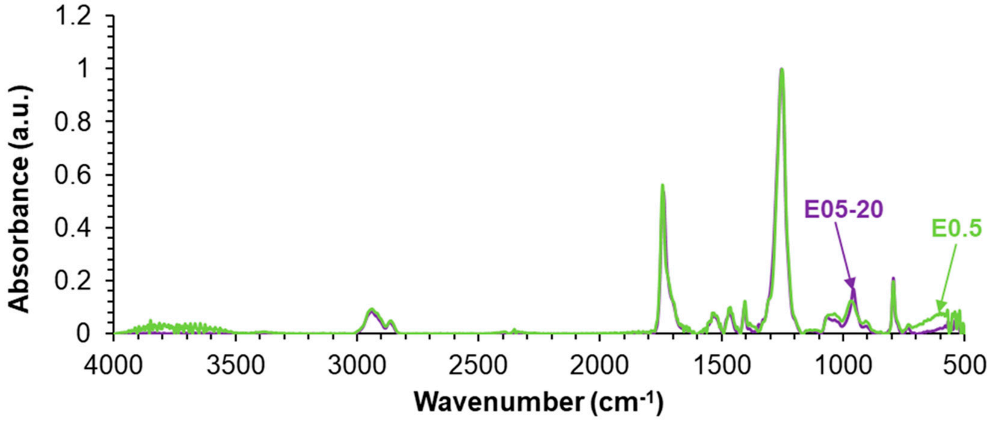

Figure S4. ATR-IR spectra of E0.5 and E0.5-20.

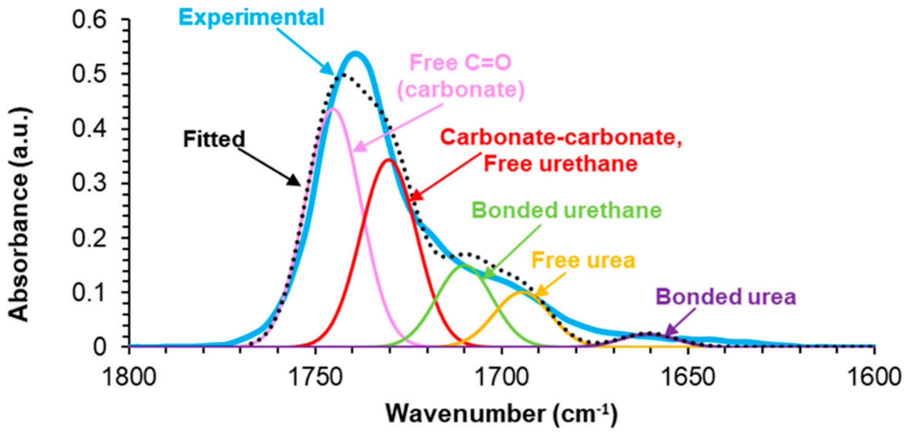

Figure S5. Curve fitting of the carbonyl stretching region of the ATR-IR spectrum of E0.5-20.

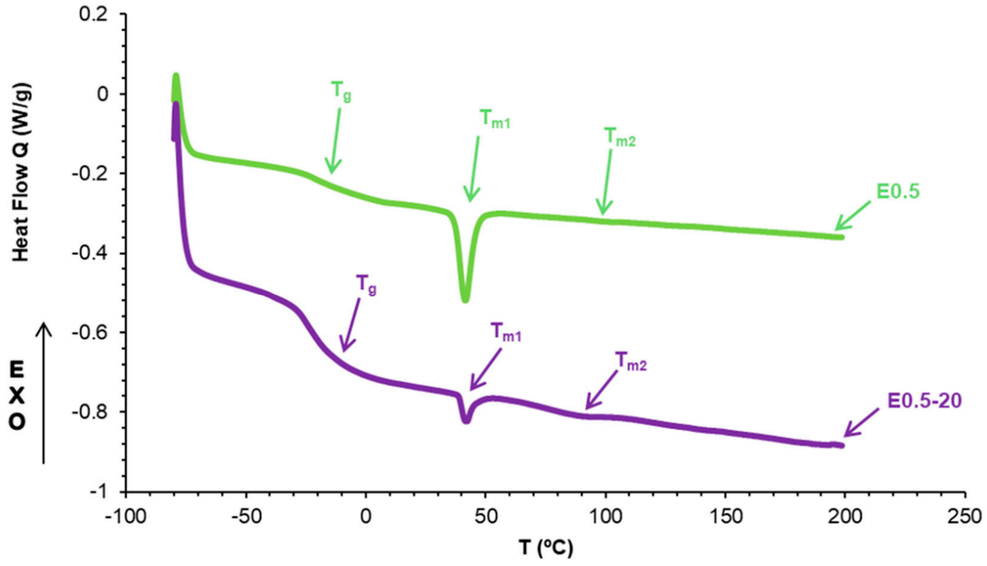

Figure S6. DSC curves of E0.5-20 and E0.5. First heating run.

Characterization of polyurethanes without and with different amounts of halloysite

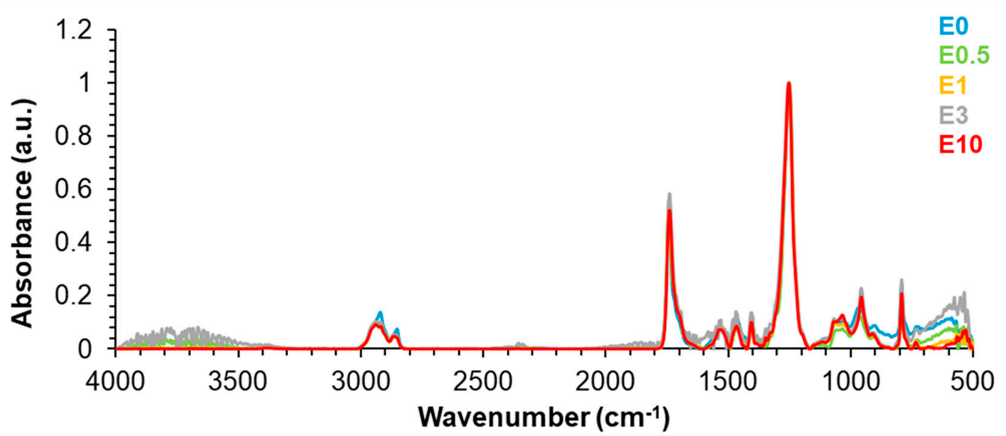

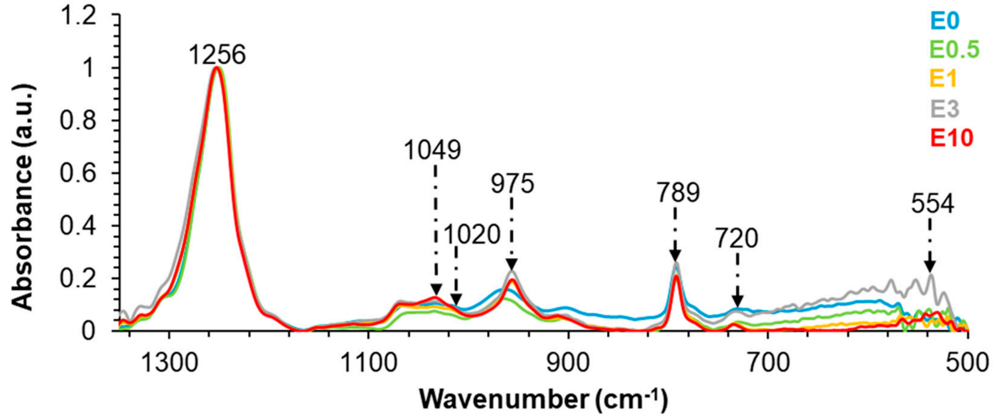

Figure S7. ATR-IR spectra of polyurethanes without and with different amounts of halloysite.

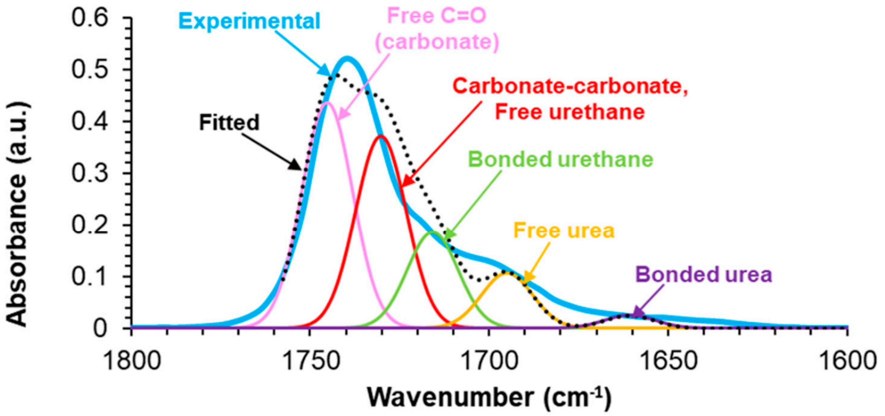

Figure S8. Curve fitting of the carbonyl stretching region of the ATR-IR spectrum of E10.

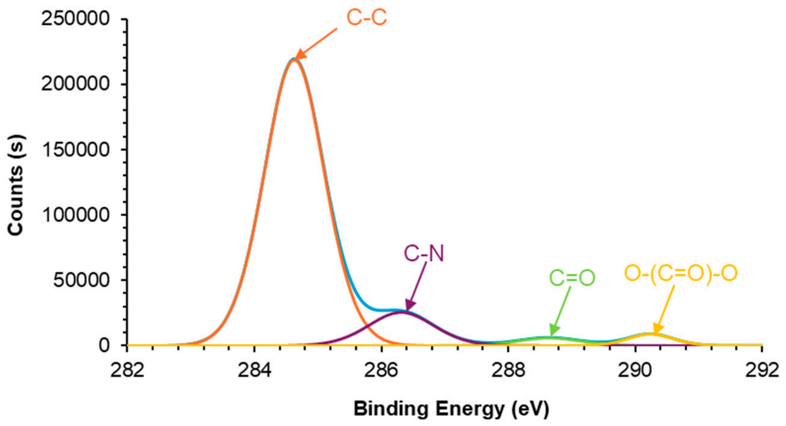

Figure S9. High-resolution C1s XPS spectrum of E0.5 surface.

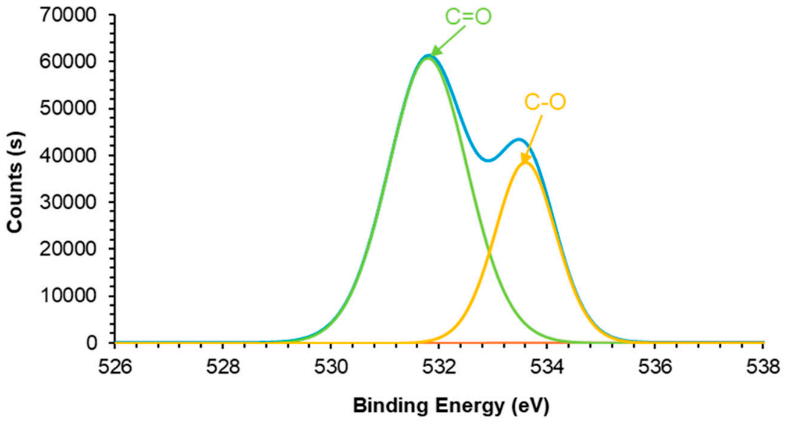

Figure S10. High-resolution O1s XPS spectrum of E0.5 surface.

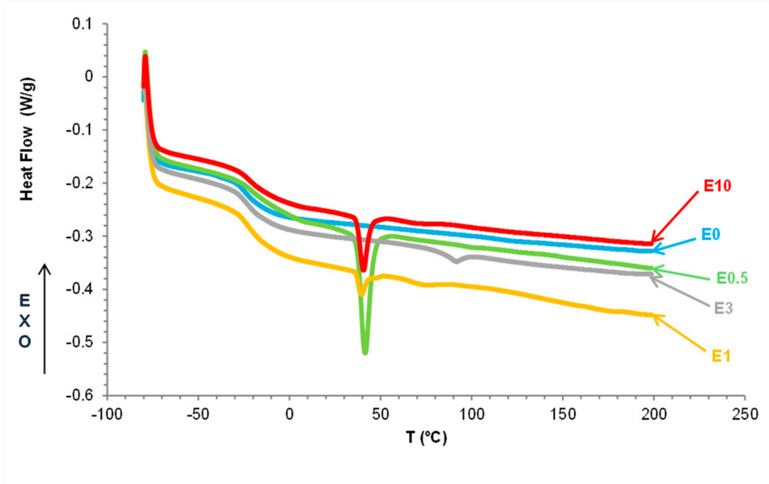

Figure S11. DSC curves of polyurethanes without and with different amounts of halloysite. First heating run.

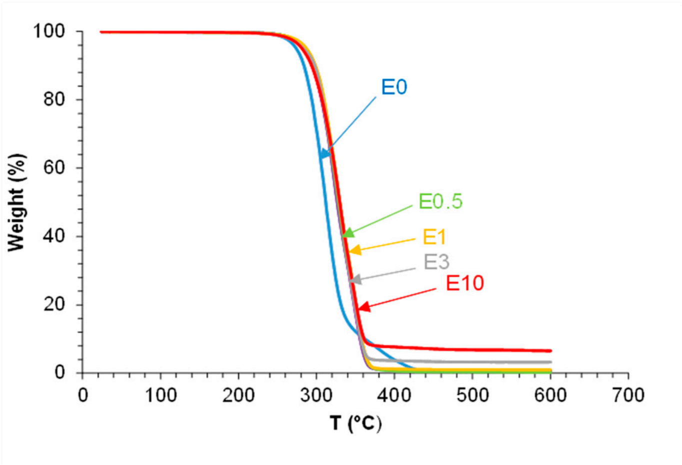

Figure S12. TGA curves of polyurethanes without and with different amounts of halloysite.

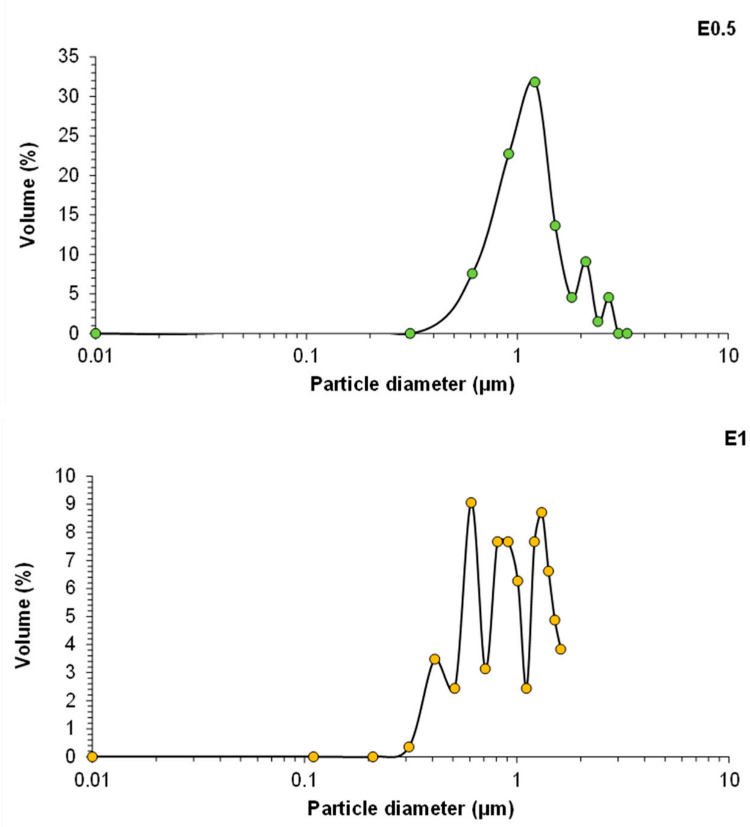

Figure 1 Halloysite particle size distributions in E0.5 and E1.

Table S2: 2θ values and intensities of the main diffraction peaks of the polyurethanes without and with different amounts of HNTs.

2θ (°)	Intensity (a.u.)				
	**E0**	**E0.5**	**E1**	**E3**	**E10**
12	-	-	-	793	782
14	1140	846	-	-	-
15	-	-	804	-	-
16	-	-	-	-	656
17	1863	-	-	-	-
18	-	-	-	-	-
19	1623	3018	-	-	-
20	3118	1918	3099	-	2408
23	2073	352	2183	2172	2172
25	772	896	-	-	-
44	416	379	408	396	396
